# The Type III Secretion System (T3SS)-Translocon of Atypical Enteropathogenic *Escherichia coli* (aEPEC) Can Mediate Adherence

**DOI:** 10.3389/fmicb.2019.01527

**Published:** 2019-07-09

**Authors:** Fernanda F. Santos, Denise Yamamoto, Cecilia M. Abe, Jack A. Bryant, Rodrigo T. Hernandes, Felipe C. Kitamura, Felipe S. Castro, Tiago B. Valiatti, Roxane M. F. Piazza, Waldir P. Elias, Ian R. Henderson, Tânia A. T. Gomes

**Affiliations:** ^1^Departamento de Microbiologia, Imunologia e Parasitologia, Escola Paulista de Medicina, Universidade Federal de São Paulo, São Paulo, Brazil; ^2^Laboratório de Bacteriologia, Instituto Butantan, São Paulo, Brazil; ^3^Institute of Microbiology and Infection, College of Medical and Dental Sciences, University of Birmingham, Birmingham, United Kingdom; ^4^Departamento de Microbiologia e Imunologia, Instituto de Biociências, Universidade Estadual Paulista, Botucatu, Brazil; ^5^Departamento de Diagnóstico por Imagem, Escola Paulista de Medicina, Universidade Federal de São Paulo, São Paulo, Brazil

**Keywords:** adherence, atypical EPEC, EspB, EspD, polymorphism, gene expression, type III secretion system (T3SS)-translocon, EspA

## Abstract

The intimin protein is the major adhesin involved in the intimate adherence of atypical enteropathogenic *Escherichia coli* (aEPEC) strains to epithelial cells, but little is known about the structures involved in their early colonization process. A previous study demonstrated that the type III secretion system (T3SS) plays an additional role in the adherence of an *Escherichia albertii* strain. Therefore, we assumed that the T3SS could be related to the adherence efficiency of aEPEC during the first stages of contact with epithelial cells. To test this hypothesis, we examined the adherence of seven aEPEC strains and their *eae* (intimin) isogenic mutants in the standard HeLa adherence assay and observed that all wild-type strains were adherent while five isogenic *eae* mutants were not. The two *eae* mutant strains that remained adherent were then used to generate the *eae*/*escN* double mutants (encoding intimin and the T3SS ATPase, respectively) and after the adherence assay, we observed that one strain lost its adherence capacity. This suggested a role for the T3SS in the initial adherence steps of this strain. In addition, we demonstrated that this strain expressed the T3SS at significantly higher levels when compared to the other wild-type strains and that it produced longer translocon-filaments. Our findings reveal that the T3SS-translocon can play an additional role as an adhesin at the beginning of the colonization process of aEPEC.

## Introduction

Enteropathogenic *Escherichia coli* strains (EPEC) are one of the leading causes of severe and even fatal childhood diarrhea in developing countries (Kotloff et al., 2013). Bacteria belonging to this pathotype are subdivided into two groups, typical EPEC (tEPEC) and atypical EPEC (aEPEC). The main difference between tEPEC and aEPEC is the lack of the EAF (EPEC adherence factor) plasmid in aEPEC (Kaper, 1996; Trabulsi et al., 2002).

Typical EPEC strains have a characteristic adherence pattern on the surface of HeLa cells, called localized adherence (LA). In LA, tEPEC adhere to the cell surface and form compact microcolonies (clusters), which can be visualized after 3 h of interaction (Cravioto et al., 1979; Scaletsky et al., 1984). This process is associated with the presence of the EAF plasmid encoding the bundle forming pilus (BFP), which belongs to the type IV fimbriae and is responsible for the binding of bacteria within microcolonies promoting their stabilization, and between microcolonies and cell surface (Girón et al., 1991; Donnenberg et al., 1992).

Since aEPEC strains are devoid of BFP, most strains produce a localized adherence-like (LAL) pattern, which is similar to LA, but with the presence of looser bacterial clusters (Rodrigues et al., 1996). In addition, other patterns of adherence may be observed among some aEPEC strains (Vieira et al., 2001; Hernandes et al., 2009; Gomes et al., 2011).

Recently, the number of infections caused by aEPEC has increased in developing and even in industrialized countries, exceeding the number of infections caused by tEPEC (Croxen et al., 2013; Donnenberg and Finlay, 2013; Kotloff et al., 2013; Foster et al., 2015; Gomes et al., 2016). Therefore, understanding how aEPEC mediate disease is necessary for the development of new control strategies (Croxen et al., 2013; Ingle et al., 2016).

Typical and atypical EPEC strains have in common the ability to produce a characteristic histopathological lesion in the intestinal epithelium termed attaching-effacing (AE) lesion (Rothbaum et al., 1982; Hernandes et al., 2009). This lesion is characterized by the intimate adherence between the bacteria and the host cell, with subsequent microvilli destruction and formation of pedestal-like structures beneath the adherent bacteria. This process results from the mobilization of different cytoskeletal proteins and actin microfilaments, which polymerize and condense at the EPEC adherence site (Rothbaum et al., 1982; Moon et al., 1983).

The genes required for the establishment of the AE lesion are located in a chromosomal pathogenicity island called LEE (locus of enterocyte effacement) (McDaniel et al., 1995). The LEE encodes the structural components of a filamentous type III secretion system (T3SS), comprising the EspA, EspB, and EspD (EPEC secreted protein) translocator proteins, effector proteins (Map, EspF, EspG, EspH, EspZ), and the proteins involved in intimate adherence. The latest include the outer membrane adhesive protein intimin, encoded by the *eae* (*E. coli* attaching and effacing) gene, and Tir (translocated intimin receptor) (Jerse et al., 1990; Kenny et al., 1997; Garmendia et al., 2005).

The T3SS-translocon comprises a filamentous structure containing a central channel through which the bacterial effector proteins pass into the host cell (Frankel et al., 1998). The connecting filament is composed by EspA, while EspB and EspD, which are located at the tip of the filament, form a pore in the eukaryotic cell membrane, allowing the injection of bacterial effector proteins (Knutton et al., 1989; Ide et al., 2001).

Tir is one of the effectors translocated to the host cell where it is inserted into the enterocyte membrane and acts as a receptor for intimin (Kenny et al., 1997). The intimin-Tir interaction is responsible for the intimate adherence of EPEC and is essential for the establishment of the AE lesion. In the absence of these proteins, tEPEC is unable to produce AE lesion, but the formation of microcolonies and bacterial adherence *in vitro* are still promoted by BFP (Cleary et al., 2004). It is well known that BFP participates in the initial stages of tEPEC interaction, prior to intimate adherence establishment and pedestal formation (Cleary et al., 2004). However, little is known about the early stages of aEPEC adherence process, since it is devoid of BFP.

The T3SS-translocon of the *Escherichia albertii* 1551-2 strain shares genetic and phenotypic similarities with aEPEC and acts as an adhesin (Hernandes et al., 2008, 2013; Yamamoto et al., 2017). Indeed, a 1551-2 intimin/EspA double mutant showed a 99.5% reduction in adherence to HeLa cells (Hernandes et al., 2013). Therefore, considering that aEPEC strains are devoid of BFP, we hypothesized that the T3SS-translocon plays a role in the early stages of colonization and in the adherence efficiency of aEPEC strains to epithelial cells. In addition, we investigated whether potential differences in the expression of the *espA*, *espB*, and *espD* genes, in the size of the T3SS-translocons and their number per bacterial cell, and polymorphisms in EspA, EspB, and EspD could be related with the adherence efficiency of aEPEC strains by the T3SS-translocon.

## Materials and Methods

### Bacterial Strains and Growth Conditions

The aEPEC strains selected for this study were previously demonstrated to be capable of forming AE lesions (Table 1). The selection criteria used were based on the adherence pattern of aEPEC strains to HeLa cells: LA_6h_ (compact clusters identified only after 6 h of infection) (Vieira et al., 2001) or LAL (Rodrigues et al., 1996), as well as their susceptibility to the antimicrobials (kanamycin, zeocin, chloramphenicol, and ampicillin), which were necessary for the mutagenesis experiments. All bacteria used in the study were stored at −80°C in Lysogeny broth (LB) and 15% glycerol. The strains were routinely cultured in LB for 18–20 h at 37°C. When necessary, appropriate antibiotics were added to the culture medium.

**TABLE 1:** Characteristics of the wild-type aEPEC strains and their derivative mutants used in this study.

**Strains**	**Characteristics**	**References**
1711-4 wt	O51:H40; LAL	Gomes et al.,2004
2012-1 wt	O26:[H11]; LAL	Gomes et al.,2004
2531-13 wt	NT:H-; LAL	Gomes et al.,2004
3522-6 wt	O129:H11; LA_6h_	Gomes et al.,2004
3881-3 wt	O63:H6; LAL	Gomes et al.,2004
4581-2 wt	NT:H40; LAL	Gomes et al.,2004
BA4095 wt	O4:H45; LAL	Abe et al.,2009
1711-4 *eae*::Kn	1711-4 carrying the suicide vector pJP5603, inserted into the *eae* gene (Nal^r^ Kn^r^)	This study
1711-4 *eae*::Kn (pINT)	1711-4 carrying the suicide vector pJP5603, inserted into the *eae* gene (Nal^r^ Kn^r^) and complemented with pINT (Clo^r^)	This study
2012-1 *eae*::Kn	2012-1 carrying the suicide vector pJP5603, inserted into the *eae* gene (Nal^r^ Kn^r^)	This study
2012-1 *eae*::Kn (pINT)	2012-1 carrying the suicide vector pJP5603, inserted into the *eae* gene (Nal^r^ Kn^r^) and complemented with pINT (Clo^r^)	This study
2012-1 Δ*escN*	*escN*::zeo (Zeo^r^)	This study
2012-1 Δ*escN* (pEscN)	*escN*::zeo (Zeo^r^)) and complemented with pEscN (Clo^r^)	This study
2012-1 *eae*::Kn Δ*escN*	2012-1 carrying the suicide vector pJP5603, inserted into the *eae* gene (Nal^r^ Kn^r^); *escN*::zeo (Zeo^r^)	This study
2012-1 Δ*espD*	2012-1 *espD*::zeo (Zeo^r^)	This study
2012-1 *eae*::Kn Δ*espD*	2012-1 carrying the suicide vector pJP5603, inserted into the *eae* gene (Nal^r^ Kn^r^); *espD*::zeo (Zeo^r^)	This study
2531-13 *eae*::Kn	2531-13 carrying the suicide vector pJP5603, inserted into the *eae* gene (Nal^r^ Kn^r^)	This study
2531-13 *eae*::Kn (pINT)	2531-13 carrying the suicide vector pJP5603, inserted into the *eae* gene (Nal^r^ Kn^r^) and complemented with pINT (Clo^r^)	This study
3522-6 *eae*::Kn	3522-6 carrying the suicide vector pJP5603, inserted into the *eae* gene (Nal^r^ Kn^r^)	This study
3522-6 *eae*::Kn (pINT)	3522-6 carrying the suicide vector pJP5603, inserted into the *eae* gene (Nal^r^ Kn^r^) and complemented with pINT (Clo^r^)	This study
3881-3 *eae*::Kn	3881-3 carrying the suicide vector pJP5603, inserted into the *eae* gene (Nal^r^ Kn^r^)	This study
4581-2 *eae*::Kn	4581-2 carrying the suicide vector pJP5603, inserted into the *eae* gene (Nal^r^ Kn^r^)	This study
4581-2 *eae*::Kn (pINT)	4581-2 carrying the suicide vector pJP5603, inserted into the *eae* gene (Nal^r^ Kn^r^) and complemented with pINT (Clo^r^)	This study
BA4095 *eae*::Kn	BA4095, carrying the suicide vector pJP5603, inserted into the *eae* gene (Nal^r^ Kn^r^)	This study
BA4095 *eae*::Kn (pINT)	BA4095 carrying the suicide vector pJP5603, inserted into the *eae* gene (Nal^r^ Kn^r^) and complemented with pINT (Clo^r^)	This study

*LA_6h_, localized adherence pattern, identified after 6 h of interaction with HeLa cells; LAL, localized adherence-like pattern, identified after 6 h of interaction with HeLa cells; NT, non-typable O antigen; Nal^*r*^, nalidixic acid resistant; Kn^*r*^, kanamycin resistant; Clo^*r*^, chloramphenicol resistant; wt, wild-type, strains of aEPEC isolated from children up to 5 years of age; pINT, pACYC184 expression vector carrying the *eae* gene derived from tEPEC strain E2348/69; pEscN, pACYC184 expression vector carrying the *escN* gene derived from E2348/69.*

### Generation of Isogenic Mutants and Complemented Strains

The *eae* mutants were obtained by using a non-polar knockout protocol (Penfold and Pemberton, 1992) with the suicide vector pJP5603 carrying a conserved region of the *eae* gene (924 pb) (pRT2), constructed by Hernandes et al. (2008) (Table 2). In this recombination process, the ORF DNA sequence of the *eae* gene was changed without disrupting the transcription of the adjacent genes. To confirm the mutagenesis, absence of intimin production was detected by immunoblotting using monospecific polyclonal antibody against the conserved region of the protein (Int 388-667) (Menezes et al., 2010).

**TABLE 2:** Plasmids and strains used in mutagenesis and complementation assays.

**Plasmid and strains**	**Characteristics**	**References**
pINT	Expression vector pACYC184 carrying the *eae* gene derived from E2348/69 (originally termed pCVD438); Clo^*r*^	Donnenberg and Kaper,1991
pEscN	Expression vector pACYC184 carrying the gene *escN* derived from E2348/69; Clo^*r*^	Gauthier et al.,2003
pRT2	Suicide vector pJP5603 carrying a conserved region of *eae* gene (924 pb), from the *Escherichia albertii* 1551-2 strain; Kn^*r*^	Hernandes et al.,2008
pKOBEG-Apra^*r*^	Plasmid containing the lambda red operon and apramycin resistance gene	Chaveroche et al.,2000
MC4160-maltΔ224::zeo-(F+)	Bacterial strain which possess the zeocin resistance cassette	Provided by J. M. Ghigo

*Apra^*r*^. resistant to apramycin; Clo^*r*^, resistant to chloramphenicol; Kn^*r*^, resistant to kanamycin.*

Mutagenesis of the *escN* gene (which encodes an essential ATPase for T3SS-assembly) and the *espD* gene (which encodes an T3SS-translocon protein) were performed by homologous recombination using the Lambda Red system described by Datsenko and Wanner (2000), with modifications. The *escN*-Zeo-F/*escN*-Zeo-R and *espD*-Zeo-F/*espD*-Zeo-R primers were constructed with 40 bases identical to the gene of interest, in the 5′ region, and with 20 bases identical to the zeocin resistance gene, in the 3′ region (Table 3). These primers were used in PCR reactions for amplification of the 499 bp zeocin resistance gene using the MC4160-maltΔ224::zeo-(F+) as template (Table 2). To confirm the *escN* and *espD* gene mutations, PCRs were performed with the primers *escN*-verf5-F/*escN*-verf3-R (Table 3), which anneal to a site external to the regions of identity, and *espD*RT-F/*espD*RT-R (Table 3), which anneal to an internal region of the gene. Gene complementation was achieved by transformation of the mutant strains with the pINT (originally termed pCVD438) and pEscN plasmids (Table 2), respectively, by electroporation. The complementation was confirmed by PCR using primers that amplify an internal region of these genes (Tables 1, 3) and by the reestablishment of the adherence phenotype.

**TABLE 3:** Primers used in the *eae*, *escN*, and *espD* mutagenesis protocol.

**Primers**	**Sequences**	**References**
*eae*-F *eae*-R	5′ CCCGGCACAAGCATAAGCTAA 3′ 5′ ATGACTCATGCCAGCCGCTCA 3′	Gannon et al.,1993
*eae*2012-F e*ae*14-R	5′ TCTAACTCATTGTGGTGGAGC 3′ 5′ CCCCATTCTTTTTCACCGTCG 3′	This study
*escN*-Zeo*-*F *escN*-Zeo*-*R	5′ TGGGAATAATATCGAACTTAAAGTATTAGGAA CGGTAAATGGTCATCGCTTGCATTAGAAAG 3′ 5′ CGCTCTGCTTTTACGAATAGATAAAATTCTGTCC AACATATTCAGAATGATGCAGAGATGTAAG 3′	Sampaio et al.,2014
*espD*-Zeo*-*F *espD*-Zeo-R	5′ ATGCTTAATGTAAATAGCGATATCCAGTCTATGAGG TCTGGGGCCAGCGCTGCTACGGCTGTCATCGCTTGC ATTAGAAAG 3′ 5′ TTAAACTCGACCACTAACAATACGGCTATTTACTCG TGCTGAGTCGGATATTAACTGAGAGAATGATGCAGAG ATGTAAG 3′	This study
*escN*-verf5-F *escN*-verf3-F	5′ TCAGGCGCTATGTGAAGAAA 3′ 5′ TACGCCTGCTTAGAGGCAAT 3′	Sampaio et al.,2014
*escN*-F *escN*-R	5′ TCAGGCGCTATGTGAAGAAA 3′ 5′ TACGCCTGCTTAGAGGCAAT 3′	Sampaio et al.,2014

### Bacterial Growth Curve

Single colonies of wild-type and mutant strains of aEPEC, in triplicate, were used to inoculate LB broth and cultured at 37°C for 18–24 h. Each culture was then sub-cultured into fresh preheated LB at a dilution of 1:50, and incubated under continuous agitation (250 rpm) at 37°C. The optical density was monitored at 30-min intervals for 6 h.

### Adherence Assays

HeLa cells (ATCC^®^ CCL-2) were routinely cultivated in plastic bottles at 37°C in 5% CO_2_ atmosphere in DMEM (2 g/L sodium bicarbonate, 4.5 g/L glucose) containing 15 mM HEPES (Sigma, St. Louis, MO, United States), 1% penicillin/streptomycin (GiBCO, Life Technologies, Carlsbad, CA, United States) and 10% fetal bovine serum (FBS, GiBCO). For the assays, cell suspensions were inoculated (1 × 10^5^ cells per well) in 24-well plates containing 13 mm diameter glass coverslips and cultured for 48 h to obtain 80% confluence (incomplete cell monolayers), under the same culture conditions described above.

Mutant strains of aEPEC were analyzed for the maintenance of the adherence capacity in HeLa cells in relation to the wild-type strains, using the methodology described by Cravioto et al. (1979), with modifications. Bacterial strains were inoculated at 1:50 dilution in 24-well microplates containing incomplete HeLa cell monolayers in DMEM with 15 mM HEPES, 2% FBS and 2% D-mannose (to block type I fimbriae-mediated adherence). After 3 h of incubation at 37°C in normal atmosphere, microplates were washed with phosphate buffered saline (PBS) (136.9 mM NaCl, 2.7 mM KCl, 8.1 mM Na_2_HPO_4_, 1.5 mM KH_2_PO_4_) and incubated for another 3 h with fresh medium (6-h assay). After further washes, preparations were fixed with methanol at room temperature for 30 min, stained with May-Grünwald/Giemsa (Merck, NJ, United States), mounted and analyzed under immersion light microscopy. Wild-type and mutagenized strains were evaluated and compared regarding their adherence capacity. Quantitative adherence assays of aEPEC strains to cultured HeLa cells were performed after 6-h incubation as described above, followed by cell lysis in sterile distilled water (30 min at 37°C) (Miyazaki et al., 2002), serial dilutions, and spreading of the bacterial suspensions on MacConkey agar for colony forming units/mL (CFU/mL) determination.

The observed differences in adherence efficiency of wild-type and mutant aEPEC strains to HeLa cells were analyzed with Prism GraphPad ver. 5.0. Unpaired, bi-directional Student’s *t*-test was used to compare means of two strains, and One-way ANOVA followed by *post hoc* Tukey HSD Test to compare means of more than two strains.

### Genome Sequencing

DNA extraction was performed using the QIAamp^®^ DNA Mini Kit (QIAGEN), according to the manufacturer’s instructions. The genomes of seven aEPEC strains were sequenced using the MiSeq^®^ System (Illumina) platform, at the MicrobesNG – University of Birmingham. Genome assembly and annotation were performed using the software SPAdes (version: 3.9.1) and Prokka (version: 1.12), respectively (Santos et al., 2018). The reads used for genome assembly were deposited at the sequence read archive (SRA) under the accession number PRJNA490882, and the whole-genome sequences were deposited in the GenBank database under the accession numbers 2012-1 (QYVC00000000), 2531-13 (QYVB00000000), 3522-6 (QYVA00000000), 3881-3 (QYUZ00000000), 4581-2 (QYYF00000000), and BA4095 (QYUV00000000). For the 1711-4 strain, only the sequences of the *espA*, *espB*, and *espD* genes were deposited in the GenBank database under the accession numbers MK465192, MK465193, and MK465194, respectively.

### Measurement of Gene Expression

To measure *espA*, *espB*, and *espD* expression, the aEPEC strains were grown overnight in LB broth, diluted (1:100) in DMEM and incubated at 37°C under constant rotation (250 rpm) until reaching an OD_600_ ≅ 1. The total RNA of each strain was then extracted using TRIzol (Thermo Fisher, TX, United States), and subsequently purified and treated with DNAse I using the Ambion^®^ RiboPure^TM^ – Bacteria (Thermo Fisher) kit. The preparations were quantified by spectrophotometry at 260 nm (NanoDrop^TM^, MA, United States) and stored at −80°C. PowerUp^TM^ SYBR^®^ Green Master Mix (Thermo Fisher) kit was used to obtain and amplify cDNA from the extracted RNA, following the manufacturer’s recommendations. Primers used for amplification were designed based on the sequence of the aEPEC strains studied (Table 4). Three quantitative independent experiments were performed in triplicate using SYBR green technology in a Real Time PCR Step One light cycler (Applied Biosystems, CA, United States). Amplification of the gene encoding the RNA subunit Polymerase (*rpoA*) was used as internal control in all experiments. The analysis was performed using the software provided by the manufacturer and the data were analyzed using the comparative cycle threshold (ΔΔ*C*_*T*_) method (Livak and Schmittgen, 2001). *espA*, *espB*, and *espD* expression results were compared to the aEPEC BA4095 strain that was used as reference. Student’s *t*-test was used to determine the expression differences of each strain in relation to the aEPEC BA4095 strain.

**TABLE 4:** Primers used in the qRT-PCR assays.

**Primers**	**Sequence**	**References**
*espA*-2012-F *espA*-2012-R	5′AGGCATCTAAGGAGTCAACCA 3′ 5′ TGTAATGTCATTGCGAGGATCA 3′	This study
*espA*-2531-F *espA*-2531-R	5′ TGCAACATCCGTTGCTAGTG 3′ 5′GATGCCTCATTCATATCAGCAA 3′	This study
*espB*-2012-F *espB*-2012-R	5′ AAGGCGCGAGTGATATTGCT 3′ 5′ CGCTCTCGTTGCTGTAGTCA 3′	This study
*espB*-2531-F *espB*-2531-R	5′ ACAATTGTCGCAGAGTTATCAG 3′ 5′ ACTTTCCGTTGCCTTAACC 3′	This study
*espB*-4095-F *espB*-4095-R	5′ CAAGCCGCTTCTACTTCTGC 3′ 5′ CATCAGCAGCCTCTTCAGCT 3 ′	This study
*espD*-2012-F*espD*-2012-R	5′ AGTACCGCTTTACCAACGCC 3′ 5′ CTTGCATTCTGAGTGCTGCG 3′	This study
*espD*-2531-F*espD*-2531-R	5′ CCACTCATTAGTGACGCCCTCTG 3′ 5′ GGCTTTCTGTTGTTCTTCAAGC 3′	This study
*rpoA*-F *rpoA*-R	5′ GCGCTCATCTTCTTCCGAAT 3′ 5′ CGCGGTCGTGGTTATGTG 3′	Walters and Sperandio,2006

### Secreted Protein Profile of aEPEC Strains

To perform the secreted protein profile of each aEPEC strain we first selected single colonies of wild-type strains, which were inoculated in LB broth and cultured at 37°C for 18–24 h. Each culture was then sub-cultured into fresh preheated DMEM medium (2 g/L sodium bicarbonate, 4.5 g/L glucose) at a dilution of 1/50 and incubated at 37°C with constant rotation (250 rpm) until they reached an OD_600_ ≅ 1. After 5 h at 37°C under continuous agitation (250 rpm), the secreted proteins contained in the supernatants of these cultures were then precipitated with trichloroacetic acid (10% TCA) and resolved by 12% SDS-PAGE. SeeBlue^®^ Plus2 Protein Standard (Invitrogen, CA, United States) was used as a molecular weight marker. Protein extracts were electrophoresed at 150 V for 1 h and 30 min. Subsequently, the gel was washed 3 times with distilled water for 5 min to remove excess SDS. To visualize the secreted protein profile, the gel was stained for 1 h under gentle agitation with Coomassie Brilliant Blue R-250 (BioRad, CA, United States) and subsequently rinsed for 30 min in distilled water.

### Immunogold Labeling

After growing overnight in DMEM at 37°C, bacterial cultures were centrifuged, washed with PBS and fixed with 4% formaldehyde. After fixation, preparations were washed, blocked with 0.2% BSA in PBS, and incubated overnight with rabbit anti-EspA antibody (1:10 dilution in PBS) at 4°C. Preparations were subsequently washed with PBS and incubated with goat anti-rabbit antibody labeled with 10 nm colloidal gold particles (Sigma-Aldrich) diluted 1:10 in PBS, for 3 h at room temperature. After further washings, preparations were negatively stained (or not) with 2% uranyl acetate in water, in order to facilitate counting and measurement of the EspA filaments and placed onto Formvar-coated nickel grids. After air-dried, preparations were then analyzed under transmission electron microscopy (TEM) (LEO 906E – Zeiss, Germany) at 80 kV.

### EspA, EspB, and EspD Sequence Analyses

For EspA, EspB, and EspD polymorphism analyses, the corresponding gene sequences were translated and compared with those of *E. albertii* 1551-2 and tEPEC prototype E2348/69 strains using the Clustal Omega^1^ and MView.^2^ Subsequently, SMART^3^ was used to identify the different domains of EspD. To search for possible functional effects of site-specific mutations in EspD, the SNAP2 program (Bromberg and Rost, 2007) was used. The search for translocon protein sequences (EspA, EspB, and EspD) deposited in the NCBI (National Center for Biotechnology Information) database was performed using BLASTP.

## Results

### Role of T3SS in the Initial Stage of aEPEC Adherence

Previous studies of our group showed that the T3SS-translocon of an *E. albertii* strain acts as an adhesin (Hernandes et al., 2008, 2013; Yamamoto et al., 2017). Therefore, considering the genetic and phenotypic similarities of *E. albertii* with aEPEC, we hypothesized that the T3SS-translocon may play a role in the early stages of colonization and in the adherence efficiency of aEPEC strains to epithelial cells. To investigate whether other bacterial structures, besides the adhesin intimin, could favor the interaction of some aEPEC strains with the host cells, we first generated *eae* mutants for each aEPEC strain studied. These mutant strains do not produce the adhesin intimin, which is considered the main adhesin among aEPEC isolates. Lack of intimin production by these mutants was confirmed by immunoblot assays (Supplementary Figure S1).

Mutagenesis in the *eae* gene caused five of the selected strains to lose the ability to adhere to HeLa cells (Supplementary Figure S2). Loss of adherence was not due to a general impairment of growth, as demonstrated by comparable growth rates between wild-type and *eae* mutant strains (Supplementary Figure S2). The restored adhesiveness of the complemented *eae* mutant confirmed the role of intimin in these strains (Supplementary Figure S2).

**FIGURE 1:**
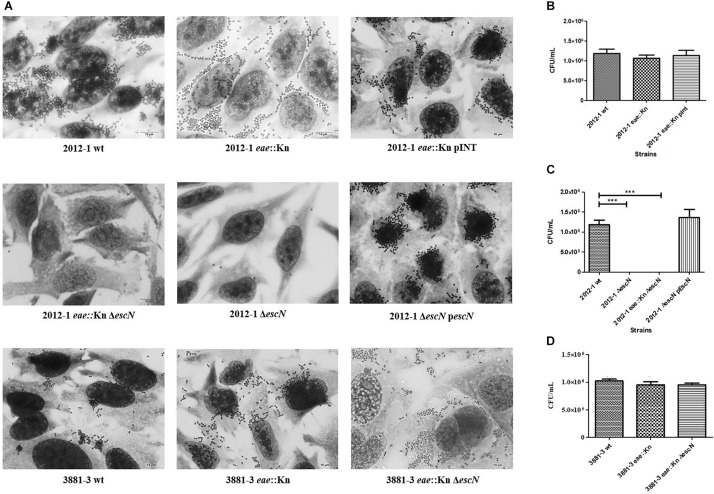
Interaction with HeLa cells of aEPEC strains 2012-1 and 3881-3 and their mutant strains, after 6 h. **(A)** Light microscopy images (microscopic magnification 1,000×) demonstrate the adherence patterns of the wild-type (wt) strains and their mutants. **(B–D)** Quantitative adherence tests. Statistical analyses of the quantitative adherence tests were performed by one-way ANOVA followed by *post hoc* Tukey HSD Test, ^∗∗∗^*p* < 0.001. The 2012-1 *eae*::Kn maintained its adherence capacity while mutants in the *escN* gene (2012-1 *eae*::Kn Δ*escN* and 2012-1 Δ*escN*) were no longer adherent. Trans-complementation of the *eae* and *escN* mutants (2012-1 *eae*::Kn pINT and 2012-1Δ*escN* p*escN*, respectively) restored the wild-type adherence phenotype. The *eae* and *escN* 3881-3 mutants (3881-3 *eae*::Kn and 3881-3 *eae*::Kn Δ*escN*, respectively) maintained the adherence capacity.

The remaining two strains (2012-1 *eae::*Kn and 3881-3 *eae::*Kn) (Table 1) maintained the ability to adhere (Figure 1). Unlike the respective wild-type strains that produced the LAL pattern, the intimin mutant strains 2012-1 *eae::*Kn and 3881-3 *eae::*Kn no longer formed clusters; instead, they spread over the cell surface (Figure 1). Growth curves in LB medium (Supplementary Figures S3, S4) and in DMEM(data not shown) confirmed that the mutagenesis procedure did not alter the bacterial growth rates. However, despite the differences found in the adherence pattern, the adherence efficiency of these mutants was quantitatively similar to that observed for the respective wild-type strain (*p* = 0.402 and *p* = 0.481, respectively) (Figures 1B,D).

Strains that remained adherent in the absence of Intimin/Tir interaction were selected, in order to verify if the T3SS-translocon could play any role in the Intimin/Tir-independent adherence. The *escN* gene, encoding the T3SS ATPase, was deleted in the wild-type and *eae*::Kn mutant backgrounds, resulting in the 2012-1 Δ*escN*, 2012-1 *eae*::Kn Δ*escN* and 3881-3 *eae*::Kn Δ*escN* mutant strains. The specificity of mutagenesis in the *escN* gene, as well as complementation of the mutant strains in *eae* and *escN*, was confirmed by PCR as shown in Supplementary Figure S5. The strains mutated in the *escN* gene did not secrete EspA, EspB and EspD, and, consequently, were not able to assemble the T3SS-translocon on their surfaces (Supplementary Figure S6).

**FIGURE 2:**
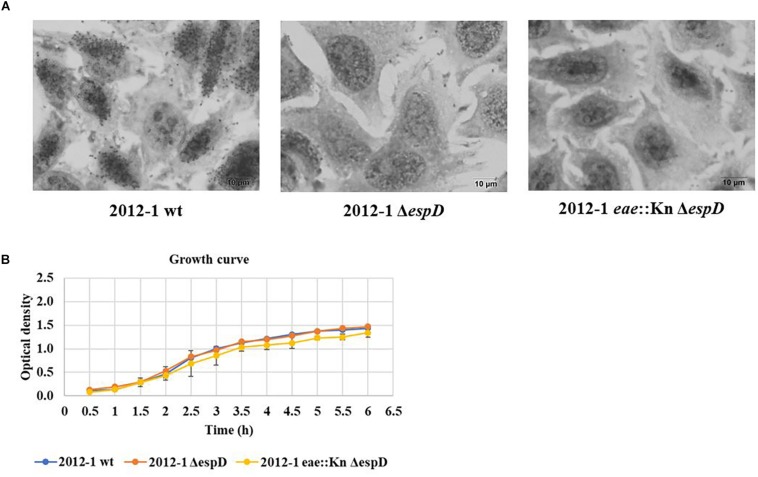
Interaction with HeLa cells of the aEPEC 2012-1 isogenic mutants in the *espD* and *eae* genes, after 6 h. **(A)** The light microscopy images demonstrate that the mutant strains totally lost their ability to adhere to HeLa cells. **(B)** The growth curves of the wild-type and the mutant strains were similar, confirming that the mutagenesis procedure did not alter bacterial growth rates.

The 2012-1 Δ*escN* and 2012-1 *eae*::Kn Δ*escN* mutants totally lost their ability to adhere, suggesting that the T3SS participates in the initial adherence steps of the wild-type strain *in vitro* (Figure 1A and Supplementary Figure S5). To confirm the role of intimin and T3SS on adherence, the *eae* and *escN* single mutants were complemented, resulting in the 2012-1 *eae*::Kn (pINT), which restored the original wild-type adherence pattern, and Δ*escN* (pEscN) strain (Table 1), which successfully restored the adherence capacity to HeLa cells (Figure 1C). Conversely, the 3881-3 *eae*::Kn Δ*escN* double mutant remained adherent, suggesting that in addition to intimin and T3SS there is probably another adhesive structure contributing to the early adherence stages of this strain (Figure 1 and Supplementary Figure S4). In addition, the 2012-1 *ΔespD* and 2012-1 *eae*::Kn *ΔespD* mutant strains were generated in order to verify if the difference found in the adherence of the wild-type strain 2012-1 and their *escN* mutant strains would be observed in the absence of EspD, the T3SS-translocon protein that interacts directly with the host cell membrane, forming the translocation pore (Knutton et al., 1989; Wachter et al., 1999; Ide et al., 2001; Chatterjee et al., 2015). As can be seen in Figure 2A, the *espD* mutant strains were no longer adherent, confirming the role of the T3SS-translocon in the adherence of strain 2012-1.The growth curves confirmed that the mutagenesis procedure and *in trans* complementation did not interfere with the bacterial growth (Figure 2B and Supplementary Figures S3, S4). Although a significant decrease was observed in the growth rates of the 2012-1 *eae*::Kn *ΔescN* and 2012-1 *ΔescN* (pEscN) mutants in LB medium, such difference was not observed in DMEM (data not shown). Furthermore, quantitative adherence assays with the latter strain showed that this decrease did not interfere with its adherence efficiency (Figure 1).

### The 2012-1 Strain Transcribes Higher Levels of *espA*, *espB*, and *espD*

A higher expression of these genes could be potentially related with a higher expression of their translated proteins and, consequently, with a higher amount of T3SS-translocons per bacterial cell or longer EspA filaments. Taking this into consideration, we could establish a relationship between the level of expression of *espA*, *espB*, and *espD* and a higher T3SS-mediated adhesiveness.

**FIGURE 3:**
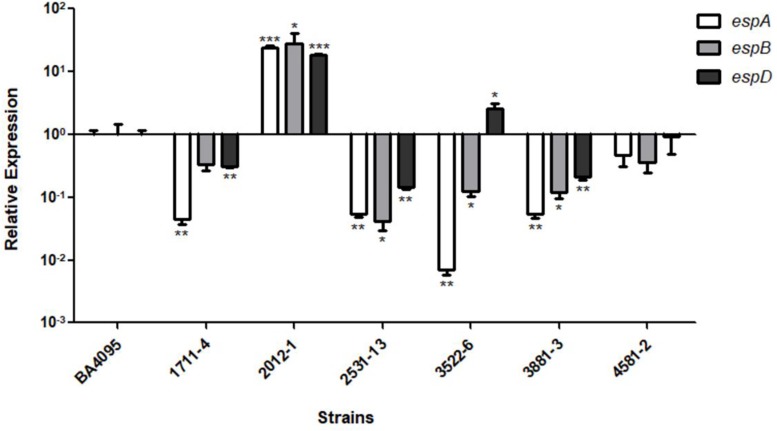
Relative expression of the *espA*, *espB*, and *espD* genes of aEPEC strains 1711-4, 2012-1, 2531-13, 3522-6, 3881-3, 4582-2 as compared to aEPEC BA4095 strain. The 2012-1 strain expresses higher levels of all three genes. Experiments were performed in biological and technical triplicates. ^*^p < 0.05, ^∗∗^*p* < 0.01, and ^∗∗∗^*p* < 0.001.

Considering this, we performed qRT-PCR assays with these genes and evaluated their expression in relation to the BA4095 strain, whose intimin mutant lost the adherence capacity (Figure 3). We showed that the relative expression of the *espA*, *espB*, and *espD* genes of the intimin-independent adherent 2012-1 strain was 23-, 26-, 18-fold, respectively, higher than the expression observed for the BA4095 strain, which relies on intimin for adherence. The remaining strains had a lower (1711-4, 2531-13, and 3881-3) or equivalent (4581-2) expression compared to BA4095. Although the expression of *espD* in the 3522-6 strain was higher than the BA4095 strain, expression of *espA* and *espB* was lower (Figure 3).

### The 2012-1 Strain Produces Longer T3SS-Translocon Filaments per Bacterial Cell

**FIGURE 4:**
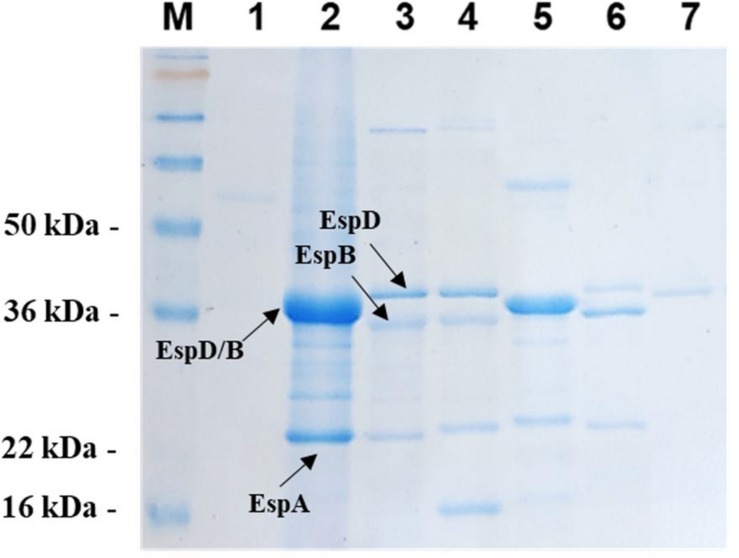
Secreted protein profile of aEPEC strains. The secreted proteins contained in culture supernatants of all aEPEC strains were precipitated, resolved by 12% SDS-PAGE and stained with Coomassie Brilliant Blue R-250. (1) 1711-4 wt; (2) 2012-1 wt; (3) 2531-13 wt; (4) 3522-6 wt; (5) 3881-3 wt; (6) 4582-2 wt; and (7) BA4095 wt. (M) SeeBlue^®^ Plus2 Protein Standard (Invitrogen).

The higher expression level of 2012-1 T3SS-translocon genes (*espA*, *espB*, and *espD*) and their respective encoded proteins (Figures 3, 4) raised the hypothesis that this strain could express higher numbers of the T3SS-traslocon proteins on the bacterial surface than the other strains, which rely upon intimin for adherence. To test this, we compared the number and length of T3SS-translocon per bacterial cell of the 2012-1 and BA4095 strains by labeling the EspA filaments.

**FIGURE 5:**
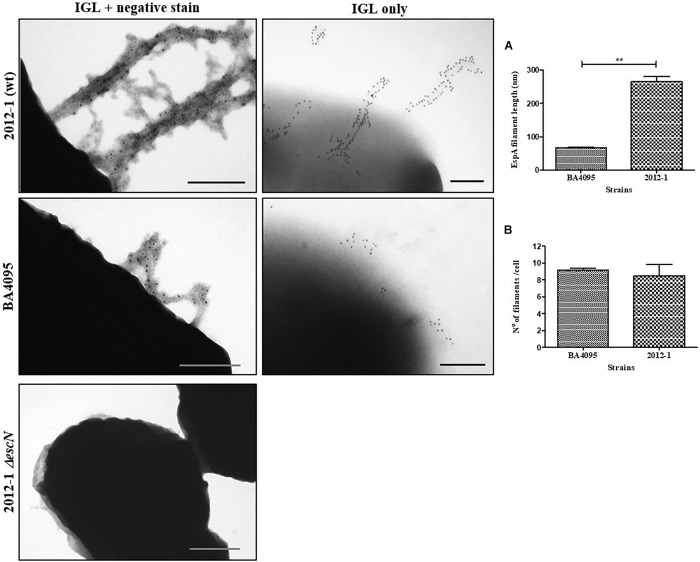
Analysis of T3SS filament formation by immunogold labeling (IGL) on aEPEC strains 2012-1 (wild-type), BA4095 (wild-type), and 2012-1 *ΔescN* (negative control). After fixation, bacterial cultures were incubated with anti-EspA antibodies, and labeled with 10 nm-gold particles. A set of preparations were firstly negatively stained with 2% uranyl acetate for general observation. In order to facilitate counting and measurement of the filaments, a second set were only immunogold-labeled. **(A)** EspA filament length and **(B)** number of filaments per bacterial cell. The strain 2012-1 produced longer EspA filaments than the strain BA4095 even though the number of T3SS-translocon per bacterial cell was the same (^∗∗^*p* < 0.01). Bars: 0.2 μm.

A total of 63 and 61 bacterial cells from the BA4095 and 2012-1 strains, respectively, were analyzed. We observed that the 2012-1 strain produced longer EspA filaments than the BA4095 strain even though, on average, the number of T3SS-translocon per bacteria cell was statistically the same between these strains (Figure 5). Supplementary Figure S7 showed that the long EspA filaments observed on the 2012-1 strain surface was also detected on the intimin mutant strain (2012 *eae*::Kn) and that nonspecific labeling was not observed in these preparations.

### The EspA, EspB, and EspD of aEPEC Strains Carry Amino Acid Regions With Significant Variations in Relation to the *E. albertii* 1551-2 and tEPEC Prototype E2348/69 Strains

Considering that the difference in amino acid sequences of the T3SS-translocon proteins could also affect the efficiency of the aEPEC strains to adhere to host cells through the T3SS, we investigated the occurrence of polymorphisms in the EspA, EspB, and EspD sequences that could be related with this phenomenon. Amino acid sequences predicted from whole-genome sequencing were aligned and showed that, although there were significant variable regions, most were conserved (Supplementary Figures S8–S10).

The EspA, EspB, and EspD sequences of the 2012-1 strain were not identical to the sequences of any other strain studied, showing between 85.0 and 88.5% identity in the EspA sequence with other aEPEC strains, 87.5% with *E. albertii* 1551-2 and 88.5% with the prototype tEPEC E2348/69 strain. Additionally, the EspB and EspD sequences of the 2012-1 strain showed 67.0 to 70.1% and 77.6 to 87.4% identity with the EspB and EspD of the other aEPEC strains, and 67.0 and 77.9% with *E. albertii*, and 70.1 and 87.4% with the tEPEC E2348/69, respectively. In contrast, the EspB sequence of the intimin-dependent adherent aEPEC BA4095 was identical to that of tEPEC E2348/69, and the EspD sequences of the BA4095, 3881-3 and E2348/69 strains were also identical.

**FIGURE 6:**
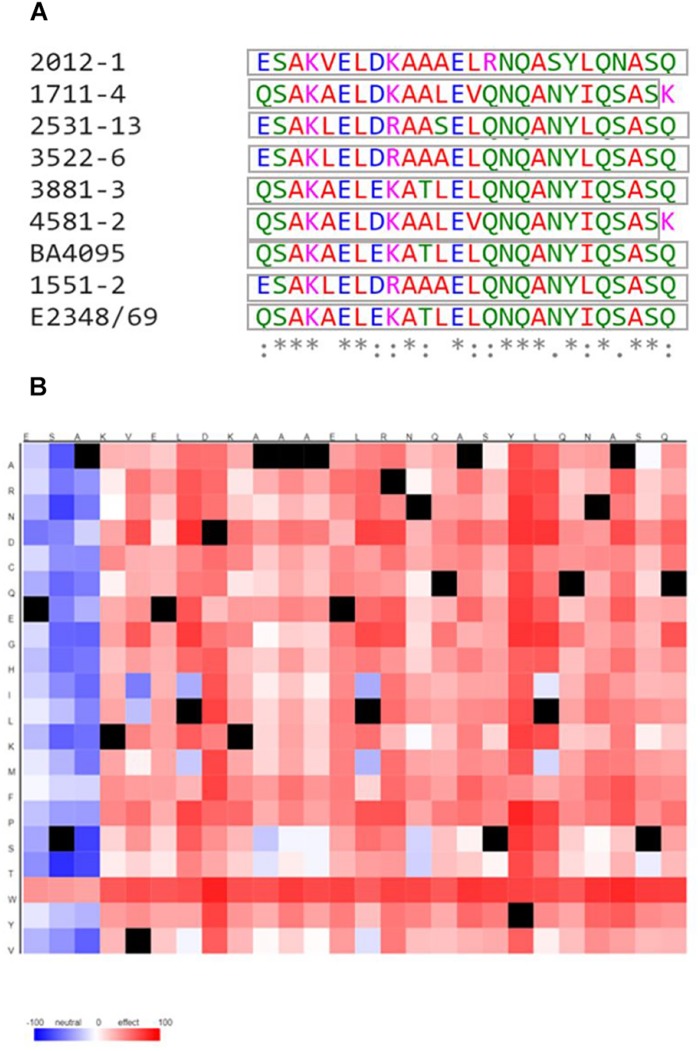
Multiple alignment among the C-terminal region of the EspD coiled-coil domain from the aEPEC strains studied and the tEPEC prototype E2348/69 strain and heat map of predicted effects of 2012-1 EspD coiled-coil domain sequence variants. **(A)** (^*^) indicates positions that have a conserved residue; (:) indicates conservation among groups of similar properties; (.) indicates conservation among groups of poorly similar properties; and (-) indicates the occurrence of gaps. The colors indicate the properties of the amino acids: (Red) small, hydrophobic and aromatic, except Y; (Blue) acids, (Magenta) basic, except H; (Green) with hydroxyl, sulfhydryl and amine groups, and Glycine (G). The gray boxes indicate the domain sequences predicted by SMART. **(B)** The colors showed are the predicted effect of each polymorphisms from the 2012-1 EspD coiled-coil domain amino acid sequence (*x*-axis) to any other amino acid (*y*-axis). Red indicates a strong signal for effect and blue a strong signal for neutral. Black marks indicate the wildtype residues.

Therefore, since differences in the amino acid sequences were found between the 2012-1 strain, whose *eae* mutant adheres by the T3SS-translocon, and each of the other aEPEC strains, we searched for polymorphisms in specific domains of EspB and EspD, by performing a comparative analysis of their functional domains. This further investigation showed that the amino acid sequences of those domains in the 2012-1 strain differed from those of the other aEPEC studied and from the *E. albertii* 1551-2 and the tEPEC E2348/69 strains (Figure 6A and Supplementary Figures S11, S12). Interestingly, in the C-terminal region of the EspD coiled-coil domain, there were amino acids that differed in their biochemical characteristics among the aEPEC strains. The 2012-1 strain had the positively charged amino acid arginine (R) at position 352, while the other strains had the uncharged amino acid glutamine (Q) in the corresponding region. This single amino acid polymorphism was predicted, by the software SNAP2 (Bromberg and Rost, 2007), to probably affect protein function (Figure 6B) suggesting, in this preliminary analysis, that specific unique or combined polymorphisms could also contribute to the adherence efficiency of the 2012-1 strain in the absence of intimin.

Alignment of the amino acid sequences of the translocon proteins (EspA, EspB, and EspD) from the 2012-1 strain to the translated genome of strains of annotated serotypes, deposited in the NCBI database, showed 100% identity with 78 *E. coli* strains of the EPEC and enterohemorrhagic *E. coli* (EHEC) pathotypes. Among these strains, there were 51 that belonged to the same serotype as strain 2012-1 (O26:H11), which had identical EspA, EspB and/or EspD proteins (Supplementary Table S1).

Among other *E. coli* strains of unidentified serotypes, 368, 293, and 229 strains were found to share 100% identity with the EspA, EspB and/or EspD sequence of strain 2012-1, respectively. Interestingly, identity with these proteins was also found among strains of *E. albertii*, ranging from 99.47% (EspD, 2 strains) to 100% (EspB, 5 strains and EspA, 7 strains).

## Discussion

Different virulence factors can be found among aEPEC strains, which have additional diversified properties (Hernandes et al., 2009; Hu and Torres, 2015; Xu et al., 2017). These factors may encode adhesive structures that contribute to a higher colonization efficiency of enterocytes, leading the host to develop a pathological condition.

In the absence of intimin, the major adhesin of AE pathogens, tEPEC strains are still able to form microcolonies and adhere to epithelial cells *in vitro* through BFP (Cleary et al., 2004). This fimbria also participates in the initial adherence stages of tEPEC, prior to the intimate adherence and consequent actin pedestals formation. However, because aEPEC are devoid of BFP, little is known about the initial stages of their adherence to the host cells.

Cleary et al. (2004) showed that an intimin/*bfpA* double mutant of the tEPEC strain E2348/69 had significantly reduced adherence capacity. On the other hand, Hernandes et al. (2013) showed that the T3SS-translocon from the *E. albertii* 1551-2 strain (formerly classified as aEPEC) (Yamamoto et al., 2017) could mediate a diffuse adherence in HeLa cells even in the absence of intimin (Hernandes et al., 2008, 2013). The 1551-2 double mutant strain in the *eae*/*espA* genes, which respectively encodes intimin and T3SS-translocon filament protein (EspA), was shown to be non-adherent in HeLa cells, indicating that the translocon plays a role in the adherence of this strain. However, further studies are required to clarify whether the EspA protein itself would act as an adhesin or the other proteins of the T3SS-translocon (EspB and EspD) would rather be responsible for the adherence of the 1551-2 intimin mutant (Hernandes et al., 2013).

In the present study, we sought to carry out a more comprehensive study on the participation of the T3SS-translocon as an adhesin in the aEPEC pathotype by analyzing *eae* (intimin) mutants of seven aEPEC serotypes. Our findings showed that five aEPEC intimin mutants lost their ability to adhere, reinforcing the importance of this protein in host cells colonization (Cleary et al., 2004; Schüller et al., 2007). However, the intimin mutants of the 2012-1 and 3881-3 strains remained adherent to HeLa cells even in the absence of intimin. In addition to maintain the ability to adhere, the adherence patterns of the 2012-1 *eae*::Kn and 3881-3 *eae*::Kn mutants were altered, as bacteria were more dispersed on the cells surfaces and no loose clusters were observed in comparison to the parent strains. Adherence pattern modification was also observed by Hernandes et al. (2013) in an *eae* mutant of the 1551-2 strain, which was attributed to the T3SS-translocon (EspA, EspB, and EspD). However, the 2012-1 *eae*::Kn and 3881-3 *eae*::Kn mutants showed no statistical difference as to their ability to adhere in relation to the wild-type strains, while despite an abundant adherence the 1551-2 *eae*::Kn mutant had a 33.7% lower adherence rate when compared to the respective wild-type strain (Hernandes et al., 2013).

Loss of adherence ability of the 2012-1 *escN* and *espD* mutants demonstrated the importance of the T3SS-translocon in the initial adherence stages of the aEPEC strain 2012-1 to epithelial cells. Furthermore, considering that EspD anchors the T3SS to the host cell membrane through the EspA filament, these findings suggested that EspD could act as the T3SS translocon-adhesin.

A strategy to increase adherence to the host cells through the T3SS machinery could be to alter the expression level of the translocon proteins EspA, EspB, and EspD. In a study of six EHEC strains that, like EPEC, carry the LEE region, bacterial adherence and LEE gene expression were compared. The strains that presented quantitatively more bacteria adhered to Caco-2 cells also expressed higher levels of *espB* and *espD*. This difference was threefold higher in relation to the less adherent strains (Kobayashi et al., 2016). Likewise, the relative expression of *espB* and *espD* of the strain 2012-1 was shown to be 26- and 18-fold higher than the BA4095, respectively. In accordance with these results, the capacity of adherence to HeLa cells of strain 2012-1 was eightfold higher than the strain BA4095 (data not shown).

These data contrast with the other aEPEC strains studied, which presented similar or reduced relative expression when compared with the BA4095 strain, except for the expression of *espD* in the strain 3522-6. The fact that the strain 3881-3, whose intimin/T3SS double mutant maintained its ability to adhere and expressed *espA*, *espB*, and *espD* in relatively lower levels than the strain BA4095, agrees with the hypothesis that there are other adhesin(s) in the strain 3881-3 mediating adherence in the absence of intimin.

Since we found significant differences in the transcription levels of *espA*, *espB*, and *espD*, we decided to proceed with the analysis of the T3SS-translocon assembly by labeling EspA in the T3SS-filament. In spite of the increased transcription of these genes in the strain 2012-1, the number of T3SS-translocons displayed per bacterial cell was similar to the number observed on the strain BA4095, which apparently depends on intimin to adhere to epithelial cells. However, the production of longer T3SS-filaments in the strain 2012-1 could favor a more efficient approach and attachment to the epithelial cells.

EspA, EspB, and EspD participate in the formation of the T3SS-translocon that allows the injection of bacterial effector proteins directly into the cytoplasm of the target cell (Knutton et al., 1989; Ide et al., 2001; Chatterjee et al., 2015). It is known that the interaction of the translocon with the host cell occurs by the binding of EspD to the cell membrane, regardless of the intimate adherence of the bacterium (Wachter et al., 1999). This observation led us to the hypothesis that this protein could contribute to the initial adherence of the strain 2012-1 to the epithelial cells.

Despite the presence of conserved regions, the EspB and EspD sequences differed among the seven aEPEC strains studied and the prototype tEPEC strain E2348/69. These results agree with the data presented in a recent study that compared the genome of 185 aEPEC strains, isolated in seven regions in Asia and Africa, and revealed 30 different LEE subtypes based on gene variation such as those of the *espA*, *espB*, and *espD* genes. In addition, these genes have specific regions that are under strong positive selection; in other words, they present a great diversity, while other more conserved regions are under strong negative selection (Ingle et al., 2016).

We showed that the sequences of the translocon proteins (EspA, EspB, and EspD) of the strain 2012-1 are different from those of the other aEPEC strains, whose intimin mutants lost the capacity to adhere to epithelial cells. These results suggest that polymorphisms in these proteins may be related to the higher adherence efficiency of the 2012-1 strain. Interestingly, based on the alignments of the 2012-1 translocon proteins with the translated genome of strains of annotated serotypes, we found that such sequences are relatively common among *E. coli* strains, including those sharing the same serotype (O26:H11) as the strain 2012-1. Therefore, these EspA,EspB and EspD specific subtypes may confer strains belonging to this serotype a greater colonization potential. However, additional studies are necessary to better understand the molecular mechanisms by which the T3SS-translocon acts favoring the adherence of some aEPEC strains.

T3SS-translocon proteins have functional domains for insertion and pore formation in the epithelial cell membrane. It has been shown that variations in amino acid composition in one or more regions of functional EspD domains among different strains could lead to changes in the efficiency of translocon-mediated adherence (Daniell et al., 2001). As mentioned above, comparative analysis showed that the EspA, EspB, and EspD of aEPEC strain 2012-1 presented different amino acid sequences compared to the other aEPEC strains studied. Interestingly, in the coiled-coil domain of the EspD C-terminal region, these amino acids differed in their biochemical characteristics and this polymorphism in the 2012-1 strain was predicted to affect protein function. This strain had the positively charged amino acid arginine (R) at position 352 of EspD, whereas the other strains possessed glutamine (Q), an uncharged amino acid, in the corresponding region. Considering that EspD plays a role in the adherence of strain 2012-1, these results suggest a potential involvement of specific amino acid sequences in the adherence efficiency of the strain 2012-1. *In vivo* studies should be performed to confirm that some subtypes of T3SS-translocons can ensure a more efficient adherence to certain strains of aEPEC, making them more competent colonizers.

In fact, Daniell et al. (2001) showed that three amino acid substitutions (Ala340Arg, Ala347Arg, Gln354Arg) of the coiled-coil domain of the C-terminal region of EspD have originated a tEPEC E2348/69 triple mutant, which preserved the ability to induce AE lesion but reduced its ability to adhere to HEp-2 cells, when compared with the wild-type strain.

Given its generalized nature and essential role in the pathogenicity of more than 25 species of Gram-negative bacteria, the T3SS is a potential target for the development of vaccines as well as new therapies with anti-virulence factors, as an alternative to the use of antibiotics in the treatment of bacterial infections. However, for such compounds to be developed, a deeper understanding of the mechanisms by which the T3SS acts in the pathogenicity of different strains is required (Keyser et al., 2008; Rasko and Sperandio, 2010; Allen et al., 2014; Zambelloni et al., 2017).

Our studies concerning the T3SS influence on the bacterial adherence to the epithelial cells *in vitro* contribute to a deeper understanding of the potential mechanisms of aEPEC virulence. The longer T3SS-translocon filaments and specific unique or combined polymorphisms in the EspA, EspB, and EspD protein could influence the efficiency of aEPEC adhesiveness. Altogether, we provided new insights for future studies regarding the T3SS-traslocon as a bacterial adhesive structure involved in host cells colonization.

## Author Contributions

TG, DY, RH, and FS conceptualized the study. FS, DY, and CA performed the formal analysis. TG and IH acquired the funding. FS and DY investigated the study. FS, DY, CA, FK, FC, RP, and TV conceived the methodology. TG administrated the project and provided the resources. TG, DY, IH, and JB supervised the study. FS validated the results. FS, TG, DY, RH, CA, IH, and JB wrote the original draft. FS, TG, DY, RH, CA, IH, JB, WE, FK, FC, and RP reviewed and edited the manuscript. All authors read and approved the final version of the manuscript.

## Conflict of Interest Statement

The authors declare that the research was conducted in the absence of any commercial or financial relationships that could be construed as a potential conflict of interest.

## Footnotes

1 https://www.ebi.ac.uk/Tools/msa/clustalo/

2 https://www.ebi.ac.uk/Tools/msa/mview/

3 http://smart.embl-heidelberg.de/#
