# Supplementary material for: The Type III Secretion System (T3SS)-Translocon of Atypical Enteropathogenic Escherichia coli (aEPEC) Can Mediate Adherence
Source: Front Microbiol. 2019 Jul 9;10:1527. doi: 10.3389/fmicb.2019.01527 (PMC6629874; doi:10.3389/fmicb.2019.01527)
Supplement: Supplementary file 1 [file Data_Sheet_1.PDF]

Supplementary Material

Supplementary Figures

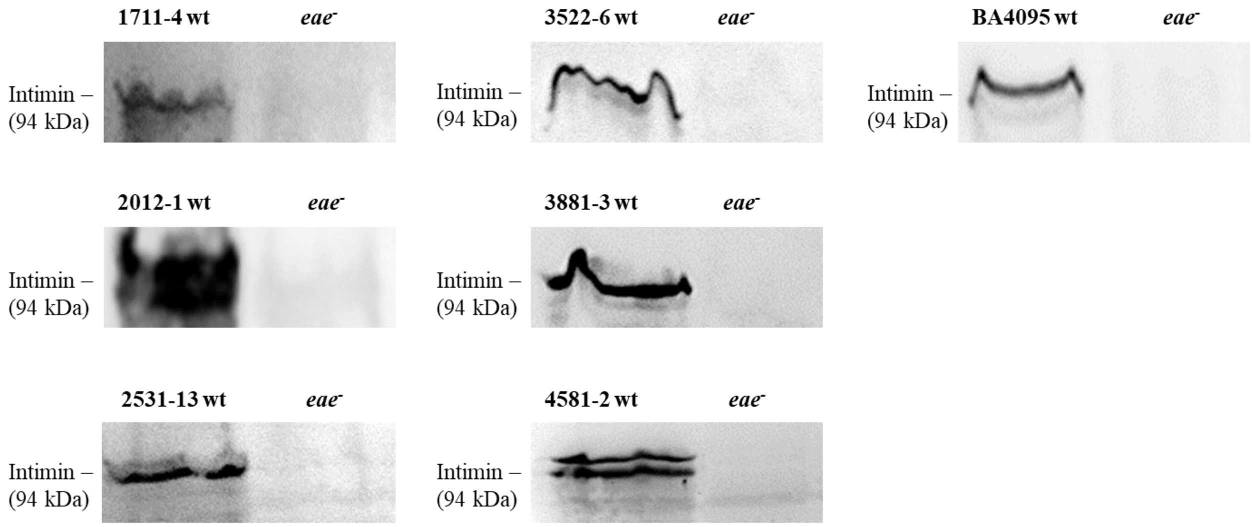

Supplementary Figure 1. Absence of intimin (~94 kDa) in the mutants in the *eae* gene of all aEPEC strains studied. Results of the immunoblotting assay of wild-type (wt) aEPEC strains with anti-intimin antibody (1:100) and respective mutants in the *eae* gene (*eae*-).

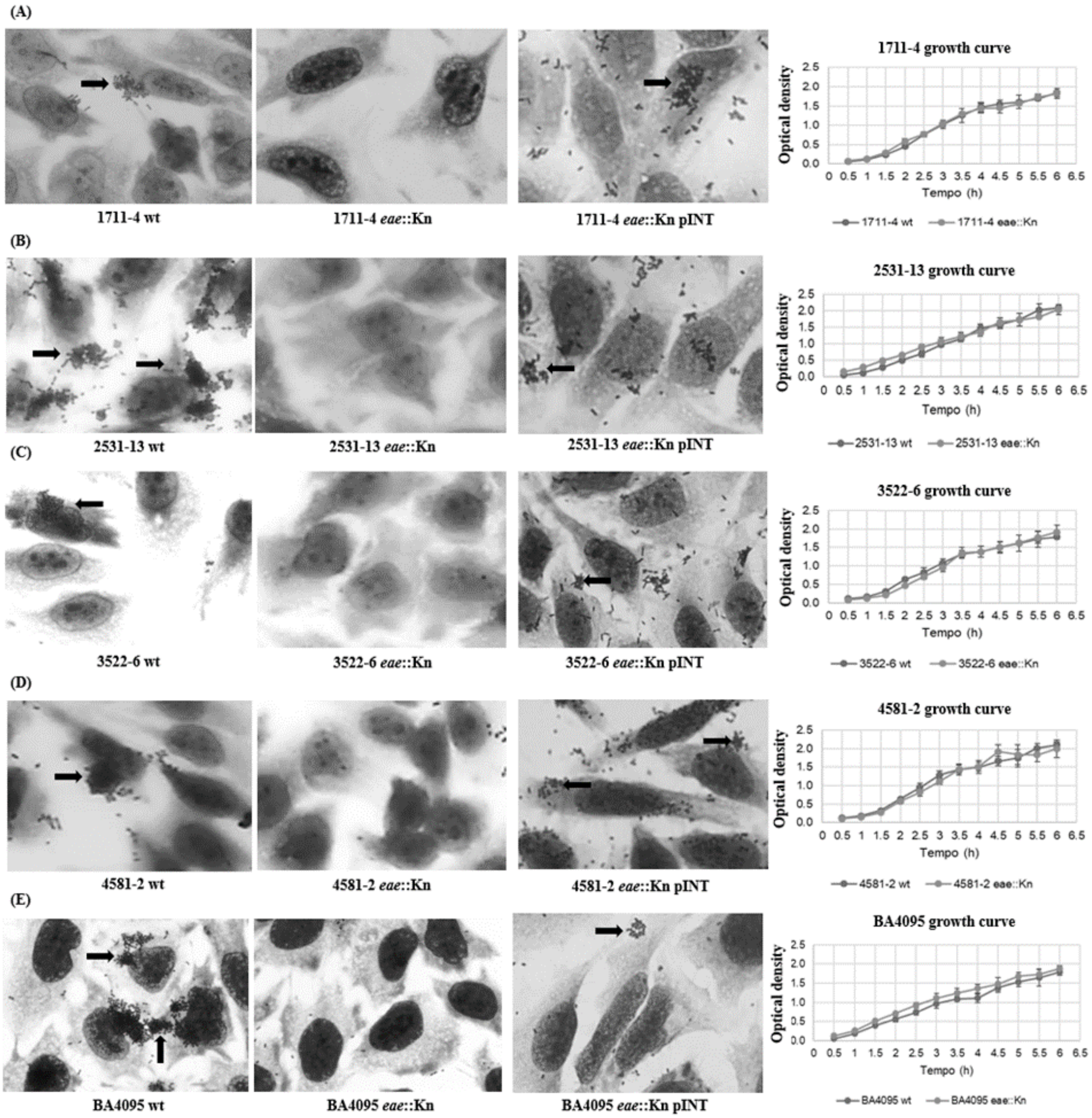

Supplementary Figure 2. Interaction with HeLa cells of five of seven selected wild-type (wt) aEPEC strains and their isogenic mutants in the *eae* gene, after 6 hours. (A-E) The light microscopy images demonstrate the adherence patterns of the wild-type (wt) strains and the absence of adherence of their respective mutant (*eae*::Kn) and complemented strains (*eae*::Kn pINT). The growth curves confirm that the mutagenesis procedure did not alter the bacterial growth rates. Microscopic magnification 1,000 x. The black arrows indicate the adherent wild-type and complemented strains.

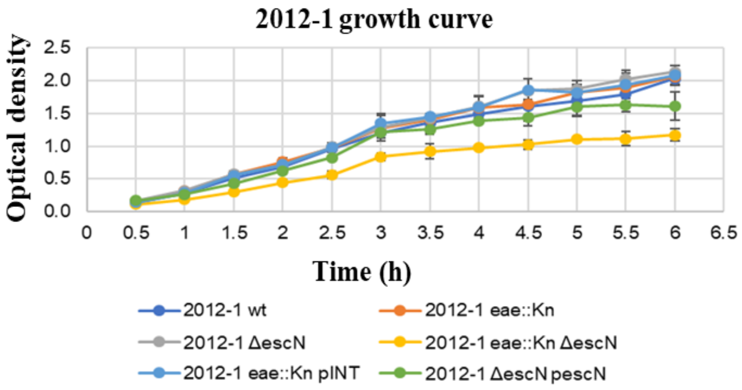

Supplementary Figure 3. Bacterial growth curves of the aEPEC strain 2012-1, isogenic mutants and complemented mutant strains. The 2012-1 growth curves of the 2012-1 Δ*escN*, 2012-1 *eae*::Kn, and 2012-1 *eae*::Kn Δ*escN* mutants and complemented mutant strains, 2012-1 *eae*::Kn (pINT) and 2012-1 Δ*escN* (p*escN*), confirm that the mutagenesis procedure did not impair bacterial growth, as demonstrated by comparable growth rates among wild-type and the mutant strains.

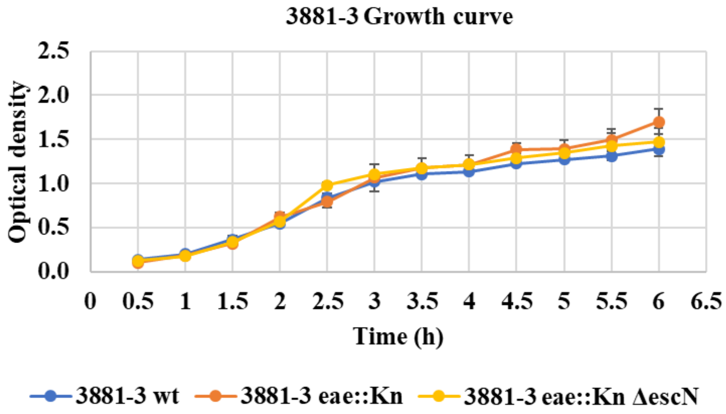

Supplementary Figure 4. Bacterial growth curves of the aEPEC strain 3881-3 and isogenic mutant strains. The growth curves of the 3881-3 *eae*::Kn and 3881-3 *eae*::Kn Δ*escN* mutants were similar, confirming that the mutagenesis procedure did not alter bacterial growth rates.

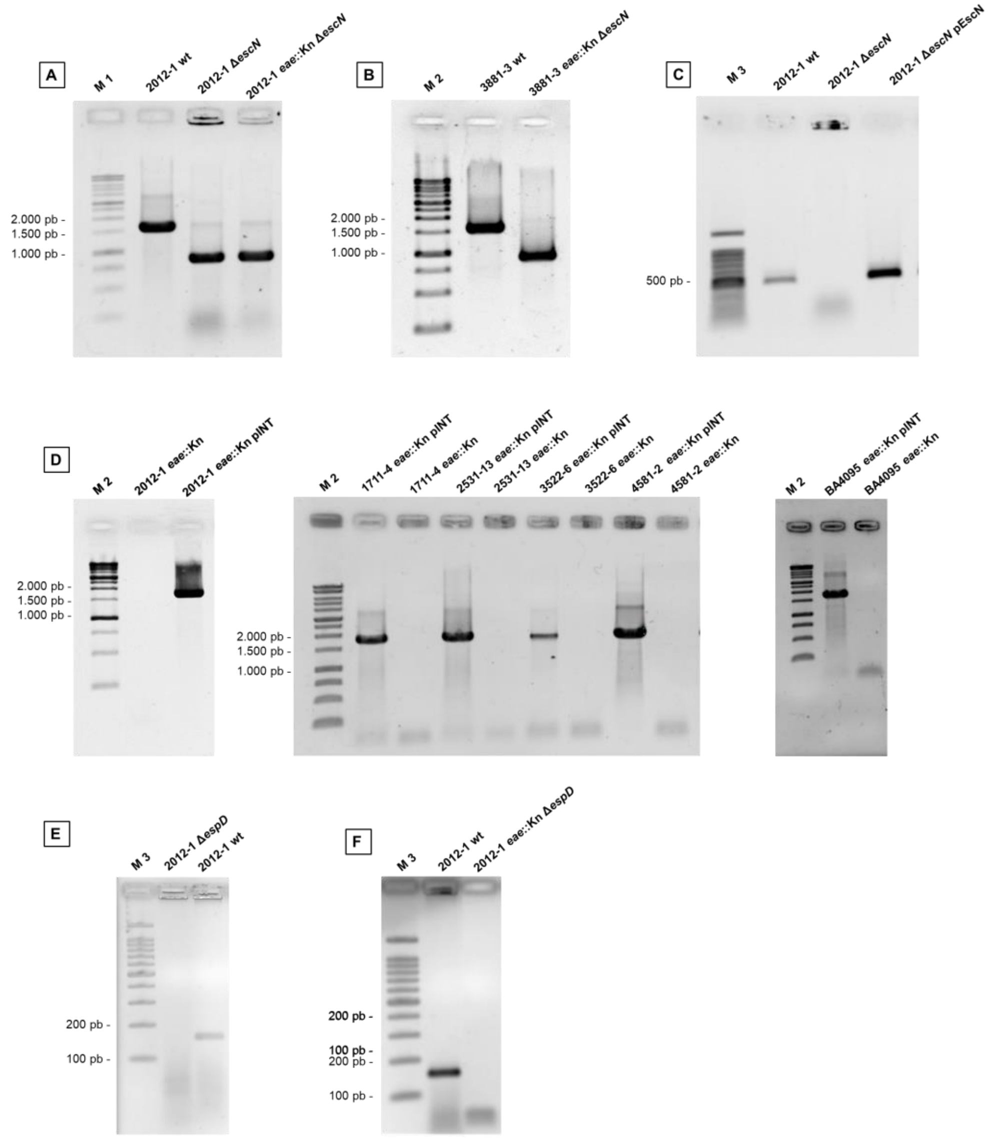

Supplementary Figure 5. Validation by PCR of the mutagenesis and complementation procedures in the *escN* and *espD* genes of aEPEC 2012-1 and 3881-3 strains. Pictures of agarose gels containing the PCR products obtained with the primers **(A)** *escN*-verf5-F/*escN*-verf3-R for strains 2012-1 wt, 2012-1 Δ*escN*, 2012-1 *eae*::Kn Δ*escN* and **(B)** 3881-3 wt and 3881-3 *eae*::Kn Δ*escN*, respectively, which amplify a DNA fragment of 1,603 bp in the wild-type strain and 889 bp in the mutant strains; **(C)** *escN*-F/*escN*-R for the strains 2012 wt, 2012-1 Δ*escN* and 2012-1 Δ*escN* pEscN, respectively, which amplify a 499 bp fragment only in wild-type and complemented strains; **(D)** *eae*2012-F/*eae*14-R, which amplify a fragment of 1,792 bp with the complemented strains (*eae*::Kn pINT); **(E)** *espD*RT-F/*espD*RT-R for strains 2012-1 wt, 2012-1 Δ*espD*; and **(F)** 2012-1 *eae*::Kn Δ*espD* that amplify a 167 bp fragment only in the wild-type strain. **(M1)**: 1 kb DNA Ladder (Promega, Wisconsin, USA); **(M2)**: 1 kb DNA Ladder (Nippon Genetics Europe, Dueren, Germany) and; **(M3)**: 100 bp DNA Ladder (Promega).

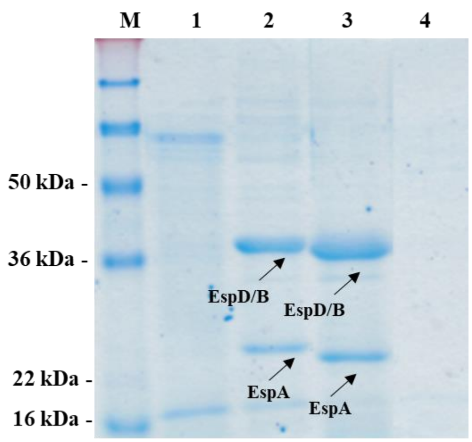

Supplementary Figure 6. Secreted protein profile of the aEPEC and *escN* mutants. The secreted proteins contained in bacterial culture supernatants were precipitated, resolved by 12% SDS-PAGE and stained with Coomassie Brilliant Blue R-250. (M) SeeBlue® Plus2 Protein Standard (Invitrogen); (1): 3881-3 *eae*::Kn Δ*escN*; (2) 3881-3 wt; (3): 2012-1wt; (4) 2012-1 *eae*::Kn Δ*escN*.

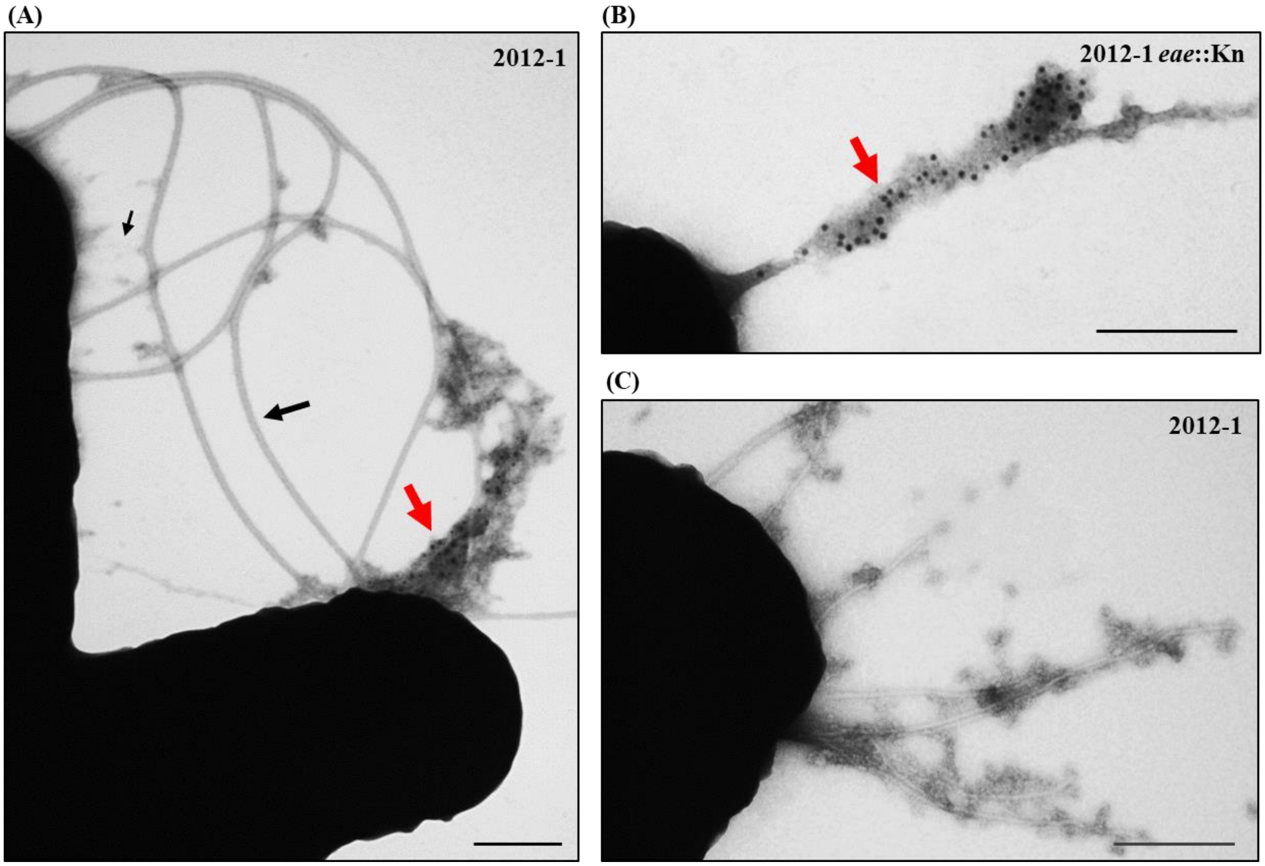

Supplementary Figure 7. Analyses of T3SS filament formation by transmission electron microscopy (TEM). After fixation, bacterial cultures were incubated with anti-EspA antibody, labeled with 10 nm-gold particles, and stained with 2% uranyl acetate. Presence of EspA labelled with gold particles (red arrow) is evident in **(A)** and **(B)**. Thinner fimbrial structures (thin black arrow) and flagella (thick black arrow) observed in **(A)** were not labeled. **(C)** shows the wild-type strain treated only with antibody labeled with gold particles, used as a negative control. Nonspecific labelling was not observed in these preparations. Bars: 0.2 µm.

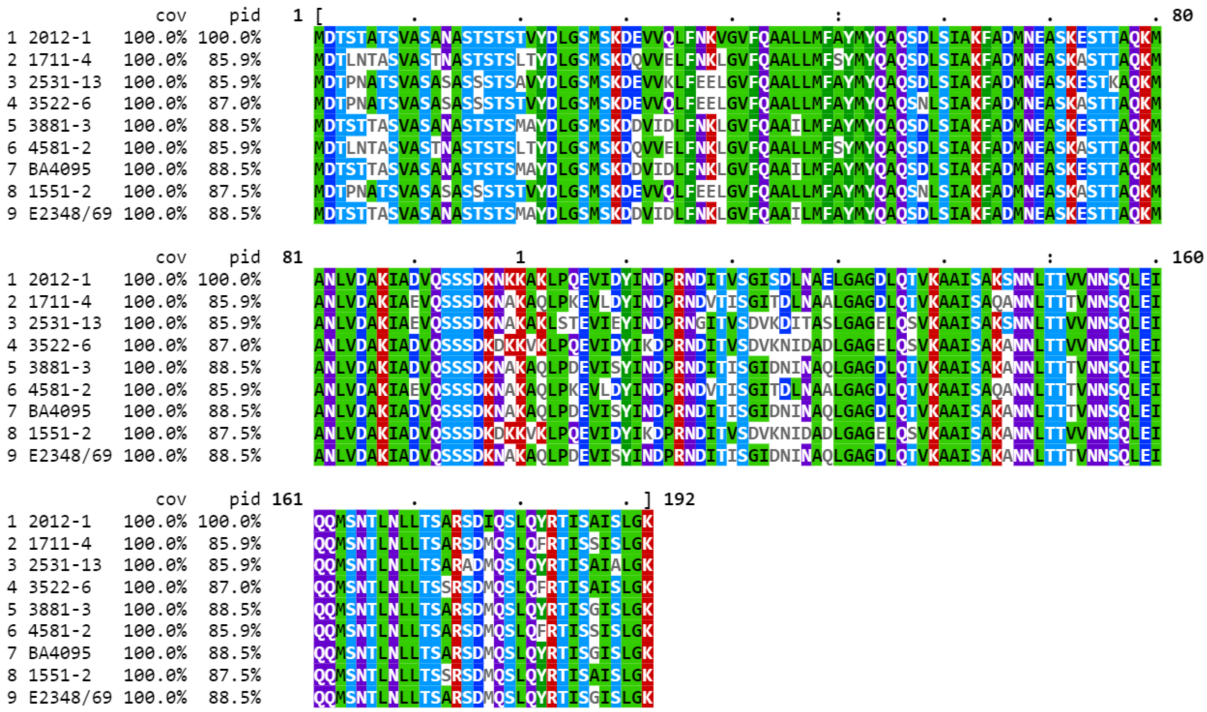

Supplementary Figure 8. Multiple alignments among the EspA sequences of the aEPEC strains, the *E. albertii* strain 1551-2 and the tEPEC prototype strain E2348/69. Reference sequence (1): 2012-1 wt. Identities normalized by aligned length. Colored by identity. (cov) covering, (pid) percentage of identity.

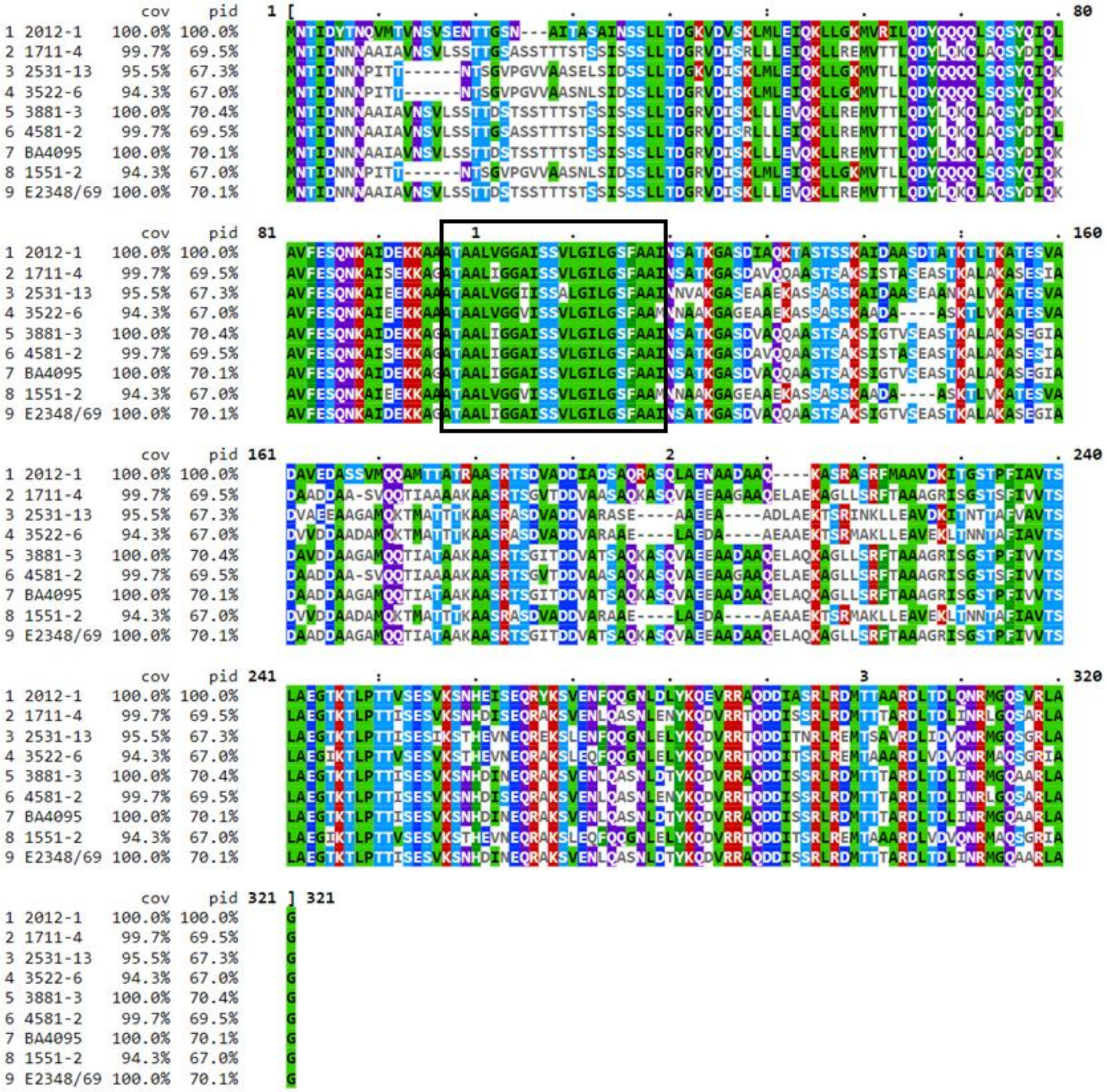

Supplementary Figure 9. Multiple alignments among the EspB sequences of the aEPEC strains, the *E. albertii* strain 1551-2 and the tEPEC prototype strain E2348/69. Reference sequence (1): 2012-1 wt. Identities normalized by aligned length. Colored by identity. (cov) covering, (pid) percentage of identity. The black square indicates the EspB transmembrane domain.

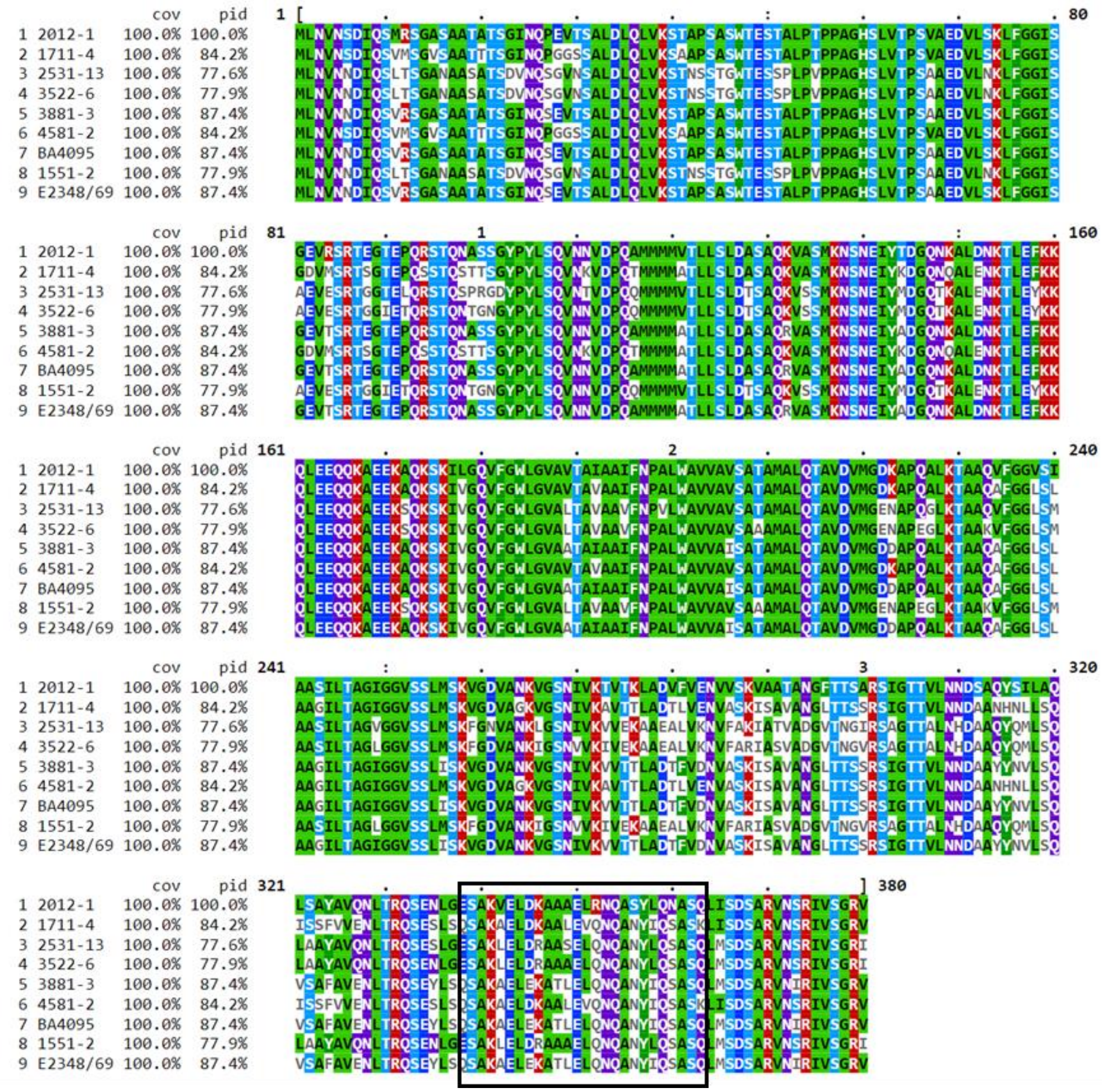

Supplementary Figure 10. Multiple alignments among the EspD sequences of the aEPEC strains, the *E. albertii* strain 1551-2 and the tEPEC prototype strain E2348/69. Reference sequence (1): 2012-1 wt. Identities normalized by aligned length. Colored by identity. (cov) covering, (pid) percentage of identity. The black square indicates the coiled-coil domain of the EspD C-terminal region.

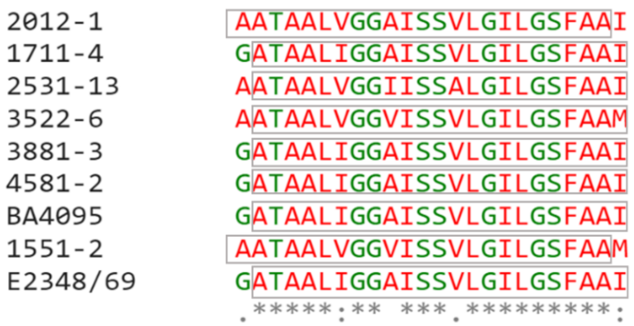

Supplementary Figure 11. Multiple alignments among the EspB transmembrane domain sequences of the aEPEC strains, the *E. albertii* strain 1551-2 and the tEPEC prototype strain E2348/69. (*) indicates positions that have a conserved residue; (:) indicates conservation among groups of similar properties; (.) indicates conservation between groups of poorly similar properties; and (-) indicates the occurrence of gaps. The colors indicate the properties of the amino acids: (Red) small, hydrophobic and aromatic, except Y; (Green) with hydroxyl, sulfhydryl and amine groups, and Glycine (G). The gray boxes indicate the domain sequences predicted by SMART.

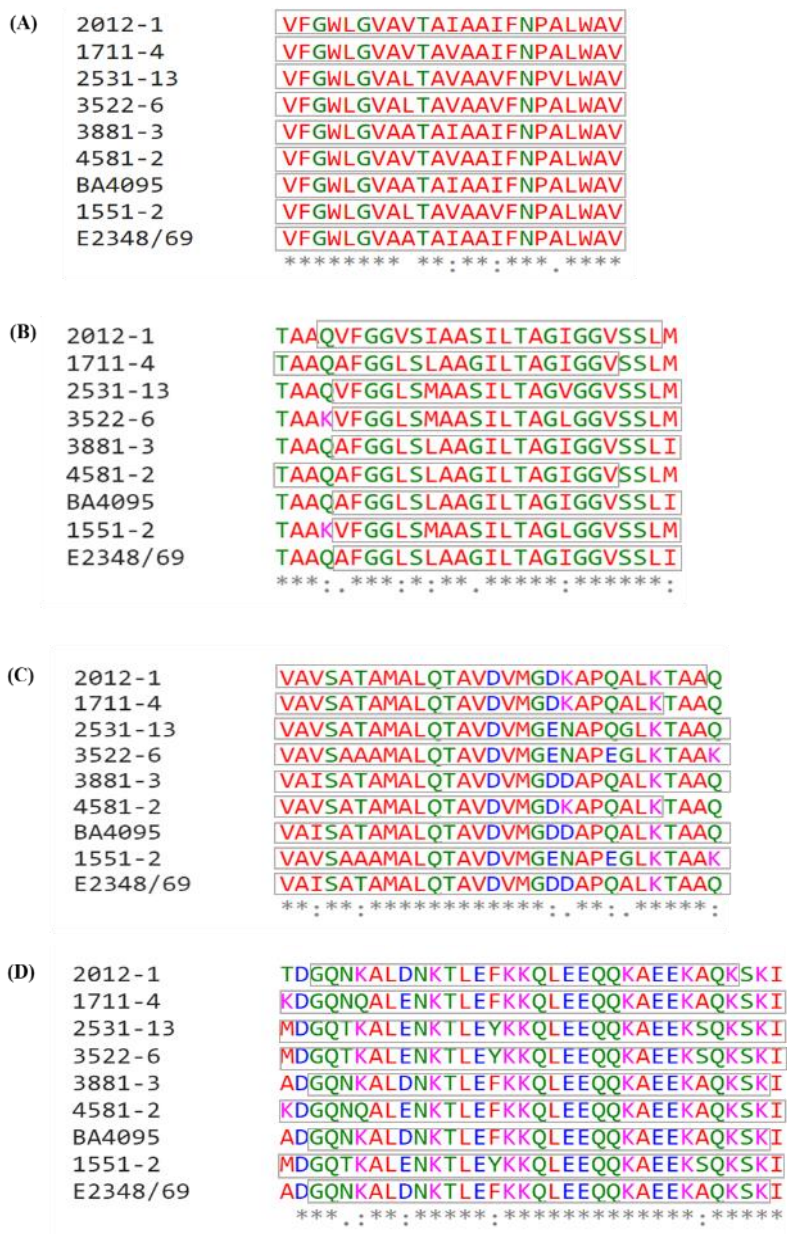

Supplementary Figure 12. **Multiple alignments among the following EspD regions**: **(A)** transmembrane domain (1); **(B)** transmembrane domain (2); **(C)** region between the transmembrane domains and; **(D)** coiled-coil domain of the aEPEC strains, the *E. albertii* strain 1551-2 and the tEPEC prototype strain E2348/69. (*) indicates positions that have a conserved residue; (:) indicates conservation among groups of similar properties; (.) indicates conservation among groups of poorly similar properties; and (-) indicates the occurrence of gaps. The colors indicate the properties of the amino acids: (Red) small, hydrophobic and aromatic, except Y; (Blue) acidic, (Magenta) basic, except H; (Green) with hydroxyl, sulfhydryl and amine groups, and Glycine (G). The gray boxes indicate the domain sequences predicted by SMART.

Supplementary Table 1. Serotypes of *E. coli* strains showing 100% identity in the amino acid sequences of EspA, EspB and EspD with those of aEPEC strain 2012-1

**Identical proteins per strain (number of strains)**	**Serotypes (number of strains)**
EspA, EspB and EspD (51)	O26:H1 (1); O26:H11 (31); O26:HNM (2); O26:HND (2); O45:H2 (1); O103 (1); O103:H11 (1); O111:H11 (3); O118:H16 (4); O123:H11 (1); O145:H28 (1); O69:H11 (3)
EspB and EspD (12)	O26:H11(4); O26:HN (1); O69:H11(1); O111:H11(1); O128:H2(3); O145:H25 (1); O177:NM (1)
EspB (5)	O26:H11 (1); O118:H16 (1); O128:H2 (1); O157:H16 (1); O165:H25 (1)
EspD (5)	O111:H2 (3); O111: HND (1); O111: HND (1)
EspA and EspB (2)	026:H11 (2)
EspA (2)	O111:H11 (2)
EspA and EspD (1)	O111:HNM (1)

The search for translocon protein sequences (EspA, EspB and EspD) of strains of annotated serotypes deposited in the NCBI database was performed using BLASTP.
